# A real-world study of the first use of palbociclib for the treatment of advanced breast cancer within the UK National Health Service as part of the novel Ibrance® Patient Program

**DOI:** 10.1038/s41416-023-02352-5

**Published:** 2023-07-19

**Authors:** Carlo Palmieri, Alison Musson, Catherine Harper-Wynne, Duncan Wheatley, Gianfilippo Bertelli, Iain R. Macpherson, Mark Nathan, Ellie McDowall, Ajay Bhojwani, Mark Verrill, Joe Eva, Colm Doody, Ruhe Chowdhury

**Affiliations:** 1 The Clatterbridge Cancer Centre NHS Foundation Trust, Liverpool, UK; 2 Department of Molecular and Clinical Cancer Medicine, University of Liverpool, Liverpool, UK; 3 The Christie NHS Foundation Trust, Manchester, UK; 4 Kent Oncology Centre, Maidstone and Tunbridge Wells NHS Trust, Kent, UK; 5 Department of Oncology, Royal Cornwall Hospitals NHS Trust, Truro, UK; 6 University Hospitals Sussex NHS Trust, Brighton, UK; 7 School of Cancer Sciences, University of Glasgow, Glasgow, UK; 8 Guy’s and St Thomas’ NHS Foundation Trust, London, UK; 9 Department of Medical Oncology, Northern Centre for Cancer Care, Freeman Hospital, Newcastle upon Tyne, UK; 10 OPEN Health, The Weighbridge, Brewery Courtyard, High Street, Marlow, UK; 11 Pfizer UK, Walton Oaks, Dorking Rd, Tadworth, UK; 12 Guys and St Thomas’ NHS Trust, Great Maze Pond, London, UK

**Keywords:** Breast cancer, Breast cancer

## Abstract

**Background:**

The Ibrance® Patient Program was established to provide access to palbociclib for UK National Health Service (NHS) patients with metastatic breast cancer (MBC), pending a funding decision.

**Methods:**

Non-interventional cohort study involving a retrospective medical record review of patients commenced on palbociclib between April and December 2017 at eight UK centres. Primary outcomes included clinicopathological characteristics, treatment patterns, clinical outcomes and selected adverse events.

**Results:**

Overall, 191 patients were identified, median age of 57.0 years (range 24.3–90.9); 30% were diagnosed with de novo MBC; 72% received first-line and 10% as ≥ second-line treatment. Median progression-free survival (95% CI) was 22.8 months (16.5–not reached [NR]) in first-line; NR in patients with de novo MBC; 7.8 months (6.8–NR) in ≥ second-line (median follow-up: 24 months). Median overall survival (OS) was NR in the overall cohort; OS rate (95% CI) at 24 months was 74.2% (67.1–81.9%) in first-line; 82.1% (72.6–92.8%) in patients with de novo MBC; 55.0% (37.0–81.8%) in ≥ second-line. Forty-seven per cent of patients developed grade 3–4 neutropenia; 3% febrile neutropenia.

**Conclusion:**

This study supports the effectiveness of palbociclib and demonstrates the benefit to patients of early access schemes that bridge the gap between regulatory approval and NHS funding for new medicines.

**Clinical trial registration:**

Clinical trial: ClinicalTrial.gov:NCT03921866.

## Introduction

Hormone receptor (HR)-positive breast cancer represents the largest subtype of the disease, accounting for over 80% of cases in the United Kingdom (UK) [1]. It has been estimated that 20–30% of women diagnosed with early-stage breast cancer will go on to develop metastatic breast cancer (MBC), and 6–10% of women have de novo MBC [2]. Endocrine therapy (ET), including aromatase inhibitors (AI), is the key treatment in the early and advanced disease setting for HR-positive breast cancer; however, a long-term clinical benefit can be limited by acquired resistance to hormonal blockade [3, 4]. The addition of a cyclin-dependent kinase 4/6 inhibitor (CDK4/6i) to an AI in the first-line setting for HR-positive, human epidermal growth factor 2-negative (HR-positive/HER2-negative) locally advanced or MBC has been shown to improve progression-free survival (PFS) [5, 6] and overall survival (OS) [7] compared to AI alone, and CDK4/6i plus AI is now considered standard of care in this setting [8, 9].

**Fig. 1:**
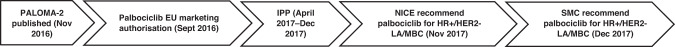
Timeline for palbociclib availability in the UK NHS. EU European Union, NICE National Institute for Health and Care Excellence, IPP Ibrance® Patient Pathway Program, SMC Scottish Medicines Consortium, HR hormone receptor, HER2 Human epidermal growth factor 2, LA locally advanced, MBC metastatic breast cancer.

Palbociclib (Ibrance®) is a CDK4/6i that was shown to be effective in HR-positive/HER2-negative locally advanced or MBC in the pivotal PALOMA clinical trials [4, 10–12] and received European Union marketing authorisation in November 2016 for HR-positive/HER2-negative locally advanced/MBC (in combination with an AI as initial ET for MBC or fulvestrant in women who received prior ET) [13] (Fig. 1). Real-world data can provide important insights into the effectiveness and safety of new therapies when used in routine clinical practice, where the patients are more heterogeneous such as being older, frailer, on concomitant therapies, or varied prognosis and in the context of palbociclib, was as supportive data alongside clinical trial data in the grant approval by the Food and Drug Administration (FDA) for the treatment of male patients with MBC in the United States of America (USA) [14, 15]. In the UK, the National Institute for Health and Care Excellence (NICE) and Scottish Medicines Consortium (SMC) approved funding for the first-line setting in November/December 2017 [16, 17]. Pending this funding approval, Pfizer made palbociclib freely available to patients in line with marketing authorisation, in combination with an AI in the first-line setting via the Ibrance® Patient Program (IPP) (April-December 2017), with a total of 843 patients from 116 National Health Service (NHS) sites accessing palbociclib. In contrast to the IPP, the prior compassionate use programme provided access prior to licencing and outside of the labelled indication in a heavily pre-treated population (see Supplementary Table 1). Reported clinical outcomes from the UK compassionate use program were a PFS of 4.5 months and an OS of 15.8 months [18].

The objectives of the present study were to describe the patient characteristics, real-world treatment patterns, clinical outcomes and selected adverse events in the first group of patients treated with palbociclib as part of the IPP in routine clinical practice.

## Methods

### Study design and setting

A UK, multi-centre, non-interventional cohort study involving a medical record review of 191 patients commenced on palbociclib (Ibrance®, Pfizer) through the IPP between April and December 2017 at eight participating NHS trusts (Liverpool, Manchester, Glasgow, Newcastle upon Tyne, Maidstone and Tunbridge, London, Cornwall, Brighton and Sussex). Study centres were selected pragmatically based on geographic representation, the number of patients enrolled in the IPP (minimum of 15 patients), and the ability to support the delivery of the study (see Supplementary methods for further details). All patients at these sites were eligible for inclusion in the study if they were aged ≥18 years at the time of enrolment into the IPP, received ≥1 dose of palbociclib as part of the IPP and gave informed consent (where required). Data were collected by trained representatives of the direct care team using electronic case report forms between February 2019 and January 2021.

### Study variables

For the present analysis we evaluated the following outcomes: clinicopathological characteristics (patient characteristics [age, sex, ethnicity, menopausal status, comorbidities]; breast cancer disease characteristics [time since initial diagnosis, recurrence, stage at time of treatment, sites of metastases, oestrogen-receptor status, progesterone-receptor status, HER2 status, and disease-free interval]; and prior breast cancer treatments); palbociclib treatment patterns (dosage, dose reductions, dosing interruptions and treatment discontinuations) and treatment duration; clinical outcomes up to 24-months post-palbociclib initiation (OS defined as the time from the date of palbociclib initiation until death from any cause as assessed by the individual centres [clinic visit, general practitioner records]; PFS defined as the time from the date of palbociclib initiation to the date of first documented disease progression or death as assessed during a clinic visit [radiological assessments]; best overall response [complete response (CR), partial response (PR), stable disease, progressive disease] as assessed by the treating physician [radiological assessments]; time to best response and time to CR/PR at 1-, 2-years post-initiation); and selected adverse events during the first 12 months post-palbociclib initiation (neutropenia, febrile neutropenia, and gastro-intestinal toxicity).

During the evaluation of patients’ breast cancer treatment history, it became apparent that not all patients received palbociclib in the first line; therefore, patients were grouped based on the treatment line as well as defined according to breast cancer treatment history for the purpose of this analysis as follows: (1) First line: patients initiated on palbociclib in combination with an AI with no prior treatment (2) First-line palbociclib added to letrozole: letrozole started >3 months prior to initiation of palbociclib. (3) Second or subsequent line: patients initiated palbociclib in combination with ET after at least one other treatment for advanced/metastatic disease.

### Statistical analyses

Quantitative variables were analysed and presented as mean (standard deviation [SD]) or median (interquartile range [IQR]; or range [minimum–maximum]), as appropriate. Categorical variables were presented as absolute and relative (%) frequency for each class. Denominators are presented where analyses were conducted in a subset of patients. Time-to-event outcomes (PFS, OS and treatment duration) were analysed and presented using the Kaplan–Meier method, with results reported as median (95% confidence intervals [CI]) and/or 12- and 24-month rates. Patients who were event-free were censored on the last date they were known to be event-free for the outcome of interest. Regarding missing data, standard imputation was used for missing dates (missing days were assumed to be the 15th of the month; missing days and month were assumed to be the 1st of July) and menopausal status (patients over the age of 60 years were assumed to be post-menopausal, 45–60 years were recorded as unknown and under 45 years were assumed to be pre-menopausal). No other imputation of missing data was conducted, and the number of patients with missing data was reported; patients with missing data were included in the calculation of percentages. All endpoints were analysed in the overall population. Subgroup analyses were conducted to describe clinical outcomes in the following subgroups: palbociclib treatment line; patients with de novo and non-de novo (relapsed) MBC; and patients with early (≤12 months) and late (>12 months) relapse (disease-free interval). The database was locked on the 4th of April 2021, and data were analysed using R studio version 3.6.1 and R studio version 1.2.1335.

## Results

### Patient demographics and clinical characteristics

A total of 191 patients (190 female [99%], 1 male [1%]) with advanced or MBC enrolled in the IPP were included in the present study. The median age at initiation of palbociclib was 57.0 years (range 24.3–90.9 years); 30% of patients were diagnosed with de novo MBC, 32% of patients had a disease-free interval of ≤12 months and 25% had a disease-free interval of >12 months; 32% had visceral involvement and 66% had non-visceral involvement with 67% of those having bone-only metastasis. Baseline patient demographics and clinical characteristics are shown in Table 1.

**Table 1:** Patient baseline demographics and clinical characteristics.

	Overall population (*n* = 191)
**Age at initiation of palbociclib (years**)^a^	
Median (range)	57.0 (24.3–90.9)
<45	32 (17%)
45–65	100 (52%)
65–75	44 (23%)
>75	15 (8%)
**Female,** ***n*** **(%)**	190 (99%)
**Ethnicity**, *n* (%)	
White	169 (88%)
Black	8 (4%)
Asian	1 (1%)
Other	1 (1%)
Missing	12 (6%)
**Diagnosed with** **de novo** **MBC at initial diagnosis**, *n* (%)	
Yes^b^	57 (30%)
No	134 (70%)
**Oestrogen-receptor status at initial BC diagnosis**, *n* (%)	
Positive	178 (93%)
Negative^c^	2 (1%)
Not available^d^	11 (6%)
**HER2-receptor status at initial BC diagnosis**, *n* (%)	
Positive^c^	1 (1%)
Negative	170 (89%)
Not available^e^	20 (10%)
**Menopausal status at initial BC diagnosis**^**f**^	
Pre-menopausal	86 (45%)
Peri-menopausal	6 (3%)
Post-menopausal	94 (49%)
Not applicable (male patient)	1 (1%)
Not available	4 (2%)
**ECOG-PS at MBC diagnosis,** ***n*** **(%)**	
0	62 (32%)
1	36 (19%)
2	14 (7%)
Missing	79 (41%)
**Disease-free interval**^**g**^, *n* (%)	
De novo metastatic disease	57 (30%)
≤12 months	61 (32%)
>12 months	48 (25%)
Missing^h^	25 (13%)
**Metastatic sites**, *n* (%)	
Visceral	62 (32%)
Non-visceral	127 (66%)
* Bone only*	85 (67%)
* Other non-visceral*	42 (33%)
Missing	2 (1%)
**Menopausal status at recurrence of disease**, *n* (% [*n* = 134])^i^	
Pre-menopausal^j^	35 (26%)
Peri-menopausal	5 (4%)
Post-menopausal	94 (70%)
**Recurrence type**, *n* (% [*n* = 134])	
Locoregional	29 (22%)
Distant	98 (73%)
Missing	7 (5%)
**Tumour (re)biopsied after MBC diagnosis**, *n* (% [*n* = 134])	
Yes	77 (57%)
No	50 (37%)
Not known	7 (5%)
**Oestrogen-receptor status at MBC biopsy**, *n* (% [*n* = 77])	
Positive	73 (95%)
Negative^c^	2 (3%)
Not known^k^	2 (3%)
**HER2-receptor status at MBC biopsy**, *n* (% [*n* = 77])	
Negative	75 (97%)
Not known^l^	2 (3%)

*ECOG-PS* Eastern Cooperative Oncology Group–performance status, *MBC* metastatic breast cancer, *BC* breast cancer, *HER-2* Human epidermal growth factor 2.

^a^Standard imputation of data was required for date of birth (191 instances [all patients only had month and year recorded].

^b^Palbociclib first line (*n* = 40), palbociclib first line added to letrozole (*n* = 10), palbociclib second line (*n* = 7).

^c^Patients were given palbociclib off label.

^d^Not available in medical records (*n* = 11).

^e^Not available in medical records due to time of initial BC diagnosis (*n* = 11); not available in medical records (*n* = 9).

^f^Standard imputation of data was used for 4 patients (3 pre-menopausal and 1 post-menopausal).

^g^Defined as time from last known date on (neo)adjuvant therapy (hormone therapy, chemotherapy) to recurrence.

^h^Treatment dates were not available due to time of initial BC diagnosis (*n* = 3); patient previously treated at a different hospital (*n* = 2); patient had no (neo)adjuvant hormone therapy (*n* = 4); no prior hormone therapy recorded (*n* = 16).

^i^Standard imputation of data was used for 5 patients (2 post-menopausal and 3 pre-menopausal).

^j^Pre-menopausal patients prescribed either LHRH or chemotherapy as per licence indication.

^k^Not available in medical records (*n* = 2).

^l^Not available in medical records (*n* = 2). Standard imputation of data was required for date of initial breast cancer diagnosis (25 instances [15 instances with only the year recorded, 10 instances with only month and year recorded]) and date of recurrent breast cancer diagnosis (13 instances [1 instance with only the year recorded, 12 instances with only month and year recorded]).

Systemic anti-cancer treatments received prior to palbociclib initiation are summarised in Table 2. Of note, 29 (15%) patients received chemotherapy for advanced disease prior to palbociclib initiation (median 1.0 [range 1.0–3.0] lines of chemotherapy). Overall, 137 (72%) patients received palbociclib with an AI as first-line therapy, 30 (16%) patients received palbociclib as first-line therapy that was added to prior letrozole (letrozole initiated a median [range] of 168 [92–3760] days prior to palbociclib), 20 (10%) patients received palbociclib with an ET as second or more lines of therapy and 4 (2%) patients were unclassified.

**Table 2:** Systemic treatments received prior to palbociclib initiation.

	Overall population (*n* = 191)
**Chemotherapy**	
Number of lines of prior chemotherapy for metastatic disease, median (range) (*n* = 29)	1.0 (1.0–3.0)
Patients treated with neoadjuvant chemotherapy, *n* (%)	
Yes	23 (12%)
No	168 (88%)
Patients treated with adjuvant chemotherapy, *n* (%)	
Yes	67 (35%)
No	124 (65%)
Chemotherapy for advanced/metastatic, *n* (%)	
Yes	29 (15%)
No	162 (85%)
**Hormone therapy**	
Number of lines of prior hormone therapy for metastatic disease, median (range) (*n* = 74)	1.0 (1.0-4.0)
Neoadjuvant hormone therapy, *n* (%)	
Yes	7 (4%)
No	184 (96%)
Adjuvant hormone therapy, *n* (%)	
Yes	110 (58%)
No	81 (42%)
Hormone therapy for advanced/metastatic, *n* (%)	
Yes^a^	73 (38%)
No	118 (62%)
Type of hormone therapy, *n* (%)	
Neoadjuvant	
* Anastrozole/letrozole*	4 (2%)
* Tamoxifen*	3 (2%)
Adjuvant	
* Anastrozole/letrozole*	33 (17%)
* Exemestane*	11 (6%)
* Tamoxifen*	84 (44%)
* Goserelin only*	2 (1%)
* Goserelin with Anastrozole*	1 (1%)
* Goserelin with Letrozole*	1 (1%)
* Goserelin with Tamoxifen, then Exemestane*	1 (1%)
Metastatic setting	
* Anastrozole/letrozole*	46 (24%)
* Exemestane*	12 (6%)
* Fulvestrant*	3 (2%)
* Tamoxifen*	12 (6%)
* Goserelin only*	2 (1%)
* Goserelin with letrozole*	9 (5%)
* Goserelin with exemestane, letrozole and tamoxifen*	1 (1%)

^a^Includes any patient, regardless of treatment line, with at least one hormone therapy recorded as being in the advanced/disease modifying/metastatic setting. Standard imputation of data was required for the date chemotherapy was started (27 instances [19 instances with only year recorded, 8 instances with only month and year recorded]) and stopped (28 instances [11 instances with only year recorded, 17 instances with only month and year recorded]); hormone therapy was started (65 instances [33 instances with only year recorded, 32 instances with only month and year recorded]) and stopped (73 instances [34 instances with only year recorded, 39 instances with only month and year recorded]).

### Palbociclib treatment patterns

Overall, 97% of patients were initiated on 125 mg/day and 3% were initiated on 100 mg/day palbociclib; 92% of patients received palbociclib in combination with anastrozole or letrozole and 7% in combination with exemestane. Palbociclib dose reductions were recorded in 41% *n* = 78) of patients and dosing interruptions in 40% *n* = 76) of patients. Palbociclib was permanently discontinued in 54% *n* = 103) of patients, most commonly due to disease progression (81% [83/103]), with 5% *n* = 5/103) discontinuing due to adverse events. The median (IQR) number of complete cycles of palbociclib was 15.0 (7.0–24.0). Median treatment duration in patients receiving palbociclib as first-line therapy was 23.5 months (95% CI 14.1–not reached [NR]). At 12 and 24 months, 65.0% (95% CI 57.4–73.5%) and 49.6% (95% CI 41.9–58.8%) of patients remained on treatment, respectively. Median treatment duration in the overall patient population was 19.3 months (95% CI: 13.9–NR). At 12 and 24 months, 62.3% (95% CI 55.8–69.6%) and 46.1% (95% CI 39.5–53.7%) of patients remained on treatment, respectively (details summarised in Table 3).

**Table 3:** Palbociclib treatment patterns during 24 months of follow-up.

	Overall population *n* = 191)
**Starting dose of palbociclib** (mg/day), *n* (%)	
125	185 (97%)
100	6 (3%)
**Dose adjustments**, *n* (%)	
Reduction	
Yes	78 (41%)
No	113 (59%)
Interruption (temporary discontinuations)	
Yes	76 (40%)
No	115 (60%)
Discontinuation	
Yes	103 (54%)
No	88 (46%)
Reasons for discontinuation, *n* (% [*n* = 103])	
* Discontinuation due to AE*	5 (5%)
* Adverse drug reaction*	3 (3%)
* Haematologic toxicity grade 3*	1 (1%)
* Non-haematologic grade 3*	1 (1%)
* Progression of disease*	83 (81%)
* Other*^*a*^	15 (15%)
**Complete cycles of palbociclib**	
Median (IQR)	15.0 (7.0–24.0)
Distribution of complete cycles, *n* (%)	
<1	5 (3%)
1–6	39 (20%)
7–12	36 (19%)
13–18	26 (14%)
19–24	52 (27%)
25–27	33 (17%)
**Duration of palbociclib treatment** (months)	
***Overall***	
Median (95% CI)	19.3 (13.9–NR)
12-month rate (95% CI)	62.3% (55.8–69.6%)
24-month rate (95% CI)	46.1% (39.5–53.7%)
***Palbociclib first line and first line added to letrozole combined*** *n* = *167)*	
Median (95% CI)	21.1 (13.9–NR)
12-month rate (95% CI)	62.9% (56.0–70.6%)
24-month rate (95% CI)	46.7% (39.7–54.9%)
***First line*** *n* = *137)*	
Median (95% CI)	23.5 (14.1–NR)
12-month rate (95% CI)	65.0% (57.4–73.5%)
24-month rate (95% CI)	49.6% (41.9–58.8%)
***First line added to letrozole*** *n* = *30)*	
Median (95% CI)	12.8 (10.4–NR)
12-month rate (95% CI)	53.3% (38.2–74.5%)
24-month rate (95% CI)	33.3% (20.1–55.3%)
***De novo MBC First line and first line added to letrozole combined)*** *n* = *47)*^*b*^	
Median (95% CI)	NR
12-month rate (95% CI)	78.7% (67.9–91.3%)
24-month rate (95% CI)	57.4% (44.9–73.5%)
**Non-*de novo MBC First line and first line added to letrozole combined)*** *n* = *120)*^*b*^	
Median (95% CI)	15.4 (11.9–NR)
12-month rate (95% CI)	56.7% (48.5–66.3%)
24-month rate (95% CI)	42.5% (34.5–52.3%)
**Endocrine partner**, *n* (%)	
Anastrozole/letrozole	175 (92%)
Exemestane	13 (7%)
Fulvestrant	2 (1%)
No recorded endocrine partner	1 (1%)
***Endocrine partner for second-line patients only, n % [n*** = ***20])***	
* Letrozole*	16 (80%)
* Exemestane*	3 (15%)
* Fulvestrant*	1 (5%)
**Ovarian suppression during palbociclib treatment,** ***n*** **(%)**^**c**^	
Goserelin	49 (26%)

*AE* adverse events, *IQR* interquartile range, *CI* confidence interval, *MBC* metastatic breast cancer, *NR* not reached.

^a^Documented reasons for discontinuation: Death *n* = 3), abdominal bloating, clinical deterioration, completed, influenza A, very poor condition, patient choice, patient moved on to have surgery, patient performance score was too low to continue, prolonged neutropenia, respiratory complications, side effects from palbociclib/bipolar disorder, subject could not swallow drug, worsening dementia symptoms (all *n* = 1).

^b^Calculated in patients given palbociclib as first line and first-line palbociclib added combined only.

^c^Defined as having goserelin at any point whilst on palbociclib.

### Palbociclib clinical outcomes

#### Overall population

**Fig. 2:**
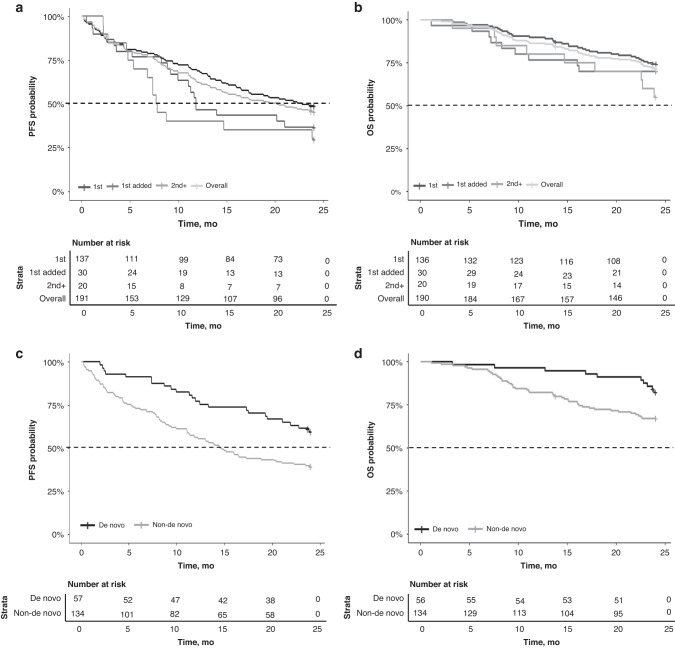
Kaplan–Meier plots of PFS and OS. PFS and OS in the overall population and by treatment line (**a** and **b**), PFS and OS in patients with and without de novo MBC (**c** and **d**). 1st: First line, no prior treatments in the metastatic setting; 1st added: First-line added to letrozole, palbociclib added to ongoing letrozole, letrozole started >3 months prior to initiation of palbociclib; 2nd+: second or subsequent line of therapy; de novo: de novo MBC patients; Non-de novo: relapsed MBC; PFS progression-free survival, OS overall survival, mo months.

At a median (range) follow-up of 24 months (1.1–24.0), the median PFS in the overall population was 20.2 months (95% CI 14.7–NR) (Fig. 2a); the 12- and 24-month PFS rates were 62.3% (95% CI 55.8–69.6%) and 45.5% (95% CI 39.0–53.2%), respectively. The median OS was not reached during 24 months of follow-up (Fig. 2b); the 12- and 24-month OS rates were 86.3% (95% CI 81.6–91.3%) and 71.5% (95% CI 65.3–78.2%), respectively. The ORR in the overall population was 42% (complete response [CR] in 2%; partial response [PR] in 40%). Stable disease occurred in 48% and progressive disease in 7% of patients (response not recorded for 3% of patients), with a median (range) time to best response of 3.5 (0.1–23.8) months *n* = 185).

#### First line

Median PFS in patients receiving palbociclib as first-line therapy *n* = 137) was 22.8 months (95% CI 16.5–NR) (Fig. 2a) and 12- and 24-month PFS rates were 68.6% (95% CI 61.3–76.8%) and 48.9% (95% CI 41.2–58.0%), respectively. The median OS was not reached during 24 months of follow-up (Fig. 2b); the 12- and 24-month OS rates were 89.7% (95% CI 84.7–95.0%) and 74.2% (95% CI 67.1–81.9%), respectively. The ORR was 45% (CR in 2%; PR in 43%). Stable disease occurred in 43% and progressive disease in 9% of patients (response not recorded for 3% of patients), with a median (range) time to best response of 3.4 (0.2–23.8) months *n* = 133).

#### First-line treatment with palbociclib added to previously initiated letrozole

Median PFS in patients receiving palbociclib as first-line therapy added to previously initiated letrozole *n* = 30) was 11.8 months (95% CI 10.0–NR) (Fig. 2a) and 12- and 24-month PFS rates were 46.7% (95% CI 31.8–68.4%) and 36.7% (95% CI 22.9–58.7%) respectively. The median OS was not reached during 24 months of follow-up (Fig. 2b); the 12- and 24-month OS rates were 76.7% (95% CI 62.9–93.4%) and 70.0% (95% CI 55.4–88.5%) respectively. The ORR was 33%. Response rates are summarised in the Supplementary results.

#### Second and subsequent line

Median PFS in patients receiving palbociclib as the second or subsequent line of therapy *n* = 20) was 7.8 months (95% CI 6.8–NR) (Fig. 2a) and 12- and 24-month PFS rates were 40.0% (95% CI 23.4–68.4%) and 30.0% (95% CI 15.4–58.6%) respectively. The median OS was not reached during 24 months of follow-up (Fig. 2b); the 12- and 24-month OS rates were 80.0% (95% CI 64.3–99.6%) and 55.0% (95% CI 37.0–81.8%), respectively. The ORR was 35%. Response rates are summarised in the Supplementary results.

#### De novo

Median PFS in the subgroup of patients with de novo MBC *n* = 57) was not reached versus 14.6 months (95% CI 11.4–22.3) in patients with relapsed MBC *n* = 134) (Fig. 2c). PFS rates at 12 and 24 months were 77.2% (95% CI 67.0–88.9%) and 59.6% (95% CI 48.1–73.8%) in patients with de novo MBC, respectively, and 56.0% (95% CI 48.2–65.0%) and 38.8% (95% CI 31.4–48.0%) for patients with relapsed MBC, respectively. Median OS in patients with de novo and relapsed MBC were not reached (Fig. 2d). OS rates at 12 and 24 months in patients with de novo MBC was 96.4% (95% CI 91.7–100.0%) and 82.1% (95% CI 72.6–92.8%), respectively, and in patients with relapsed MBC was 82.1% (9%% CI 75.8–88.8%) and 67.0% (95% CI 59.5–75.5%), respectively. Response rates are summarised in the Supplementary results.

### Selected adverse events temporally associated with palbociclib therapy

In the first 6 months following initiation of palbociclib, neutropenia (any grade) was recorded in 168 (88%) of patients (76 [40%] grade 3 and 13 [7%] grade 4). While febrile neutropenia was recorded in 6 (3%) patients during the first 12 months of treatment. Nausea, vomiting and diarrhoea (any grade) were reported in 34 (18%), 21 (11%) and 31 (16%) of patients, respectively, in the first 12 months of treatment (Table 4).

**Table 4:** Selected adverse events temporally associated with palbociclib therapy during the first 12 months post-initiation.

	Overall population *n* = 191)
**Haematologic**, *n* (%)	
***Neutropenia*** reported in the 6 months post-palbociclib initiation^a^	
Yes (All grade)	168 (88%)
Grade 3	76 (40%)
Grade 4	13 (7%)
No	23 (12%)
***Febrile neutropenia***	
Yes	6 (3%)
No	185 (97%)
**Non-haematologic**, *n* (%)	
***Diarrhoea***	
Yes (All grade)	31 (16%)
Grade 3	1 (1%)
Grade not available	9 (5%)
No	160 (84%)
***Nausea***	
Yes (All grades)	34 (18%)
Grade 1	22 (12%)
Grade 2	2 (1%)
Grade not available	10 (5%)
No	157 (82%)
***Vomiting***	
Yes (All grade)	21 (11%)
Grade 1	12 (6%)
Grade 2	1 (1%)
Grade not available	8 (4%)
No	170 (89%)

^a^Based on absolute neutrophil counts recorded in the 6 months post-palbociclib initiation, this represents the worst grade of neutropenia experienced for each patient.

## Discussion

This study reports data on the first experience of treating HR-positive/HER2-negative locally advanced/MBC patients with palbociclib within routine NHS clinical practice, where the patient populations are more heterogeneous. At the time of the IPP, no CDK4/6i was routinely available within the NHS in the first-line setting as per regulatory approval. Prior compassionate use programmes have provided access prior to licencing and outside of the labelled indication in a heavily pre-treated population [18]. The IPP, the first-ever scheme of its kind, provided access prior to reimbursement being approved by NICE and SMC, enabling first-time access to a CDK4/6i within the NHS. In total, 843 patients in 116 NHS hospitals accessed palbociclib via the IPP over a period of 8 months. In the current analysis, we report on 191 of these patients. This group of patients were of broadly similar age to those enrolled in PALOMA-2 and previous real-world studies (median 51–67 years) of patients with HR-positive/HER2-negative MBC treated with palbociclib [19–29]. In this study, 70% were considered post-menopausal, with pre-menopausal women making up 26% of the patient population at recurrence of disease, consistent with other real-world studies [20–22, 25–30]. The present study included one male; data including males (4 and 10) has been reported from two other real-world studies [19, 31].

We presented a cohort where 30% of patients had de novo MBC, which is consistent with the rate within PALOMA-2 (37.6%) [4] and within the range reported by other real-world studies of palbociclib where 11–45% had de novo MBC [20, 21, 23–25, 27–29, 32]. PALOMA-2 reported a hazard ratio of 0.67 (95% CI 0.46–0.99) for PFS in patients with de novo MBC [4]. In this study, PFS and OS were not reached at 2 years in patients with de novo MBC, whereas, in patients with relapsed MBC, PFS was 14.6 (95% CI 11.4–22.3) months and OS was not reached. PFS was previously reported as not reached (median follow-up 14.63 months) in Asian de novo MBC patients treated with palbociclib plus AI [21]. Furthermore, subgroup analysis of de novo MBC patients in the MONALEESA-2 clinical trial that combines ribociclib (an alternative CDK4/6i to palbociclib) with letrozole showed prolonged PFS, consistent that de novo MBC patients derive the best outcomes across CDK4/6i’s [33]. The beneficial clinical outcomes we report in the de novo population as compared to those treated with prior adjuvant therapies have also been observed and reported in patients with de novo HER2-positive MBC patients within the PERUSE study [34]. These results likely reflect the lack of acquired resistance as a result of prior treatment exposure.

The IPP was established to give first-line access in the advanced setting in combination with an AI as per the licensed indication in the UK; however, not all patients met these criteria, as some clinicians accessed palbociclib for their patients outside of the first-line setting. Patients receiving first-line treatment with palbociclib had a median PFS of 22.8 months (95% CI 16.5–NR), which is consistent with findings from the PALOMA-2 (24.8 months 95% CI 22.1–NR [4]) and other real-world studies of palbociclib in first-line setting from the USA (PFS range 20.0–21.2 months [19, 23, 30]) and Europe (PFS range 14.0–24.7 months [25, 28, 35]), although patient demographics and clinical practises potentially differ between these studies. Whereas in the overall population, regardless of treatment line, PFS was 20.2 months (95% CI 14.7–NR), with 18 (9%) patients recording a PFS of <2 months. Furthermore, 39 (20%) patients in the overall population continued palbociclib treatment after documented progressive disease. Further review of these 39 patients’ records revealed that this was mainly a clinical decision, with 15% of patients clinically progressing prior to starting palbociclib (Supplementary Table 4), suggesting progression from prior treatment. These results highlight the difficulty faced when using real-world scans to calculate PFS. Of note, 31% of the overall population was over 65 years; these patients are generally considered to be frailer and have shorter life expectancies with multiple comorbidities [36]; however, this did not appear to affect the efficacy outcomes in the overall study population. Furthermore, recently published data from the USA found that patients ≥65 years treated with palbociclib plus letrozole in the first line had comparable PFS of 22.2 months, suggesting that age does not affect the efficacy of palbociclib [37].

Two recently published real-world observational studies assessed OS rates in HR-positive/HER2-negative MBC patients. In the P-REALITY study of first-line patients treated with palbociclib plus letrozole, the 24-month OS rate was 78.3%, and 64.8% remained alive at 36 months [23]. Whereas in the European IRIS study, patients treated with palbociclib plus AI achieved a 24-month OS rate of 90.1%; however, it is important to note this result included patients who were > second-line [38]. The OS rates observed at 12- and 24-months (89.7% and 74.2%) post-initiation in the present study were broadly consistent with these studies and the limited data on OS reported in other real-world studies (94.5–96.5% at 12 months and 81.8–94.7% at 24 months [20, 25, 38, 39]). Median OS in the current study and in many of the previously published studies were not reached during the follow-up period; however, data from the POLARIS prospective study observed a median OS of 50.8 months in patients treated with palbociclib plus ET (median follow-up 35.7 months) [40]. Furthermore, updated data from PALOMA-2 found that palbociclib, in combination with letrozole, had an OS of 53.9 months (median follow-up of 90 months) [41]. While MONALEESA-2, which combined ribociclib with letrozole, resulted in a significantly prolonged OS (63.9 months) compared to letrozole alone (51.4 months) (median follow-up 26.4 months) [42], suggesting longer follow-up times may be required to assess OS in patients treated with CDK4/6i in combination with AI in routine clinical practice.

In those patients where palbociclib was initiated within the first-line setting >3 months (Median [range] of 168 [92–3760] days) after letrozole had been initiated, the PFS was 11.8 months (95% CI 10.0–NR), suggesting that early initiation of palbociclib with ET attain the most benefit. The use of palbociclib as a second or subsequent line of therapy with ET was associated with a median PFS of 7.8 months (95% CI 6.8–NR); this is comparable to that reported by PALOMA-3 within the second-line setting with palbociclib (9.5 months (95% CI 9.2–11.0) [11]). Of note, within PALOMA-3, palbociclib was combined with fulvestrant, while in the current study, 95% of patients received palbociclib in combination with an AI. The comparable PFS suggests that within the second-line setting, an AI can be used without compromising efficacy. Comparable clinical efficacy seen with fulvestrant and an AI in the second-line setting within the EFECT trial [43] would further support the notion that an AI or a selective oestrogen receptor down-regulator could be used as endocrine backbones with palbociclib in the second-line setting. This data is also broadly consistent with PFS observed in previous real-world studies (second-line 7.8–13.1 months; [19, 25, 28, 30, 35]).

Whilst the vast majority of patients initiated on the recommended dose of 125 mg/day palbociclib [44], dose reductions and dosing interruptions were recorded in approximately 40% of patients. These results are broadly consistent with previous studies, although the rates reported are highly variable. In the current study, 54% of patients permanently discontinued palbociclib during follow-up (24 months), 81% due to disease progression and 5% due to adverse events. Treatment discontinuation observed in previous studies ranged between 2% and 33.5% (median follow-up 6–36 months) [20, 22, 24, 25, 29, 30], with disease progression and adverse events being the main reasons for discontinuation [22, 25]. Furthermore, the frequency of selected grade 3 and 4 adverse events, including febrile neutropenia, in our study was consistent with expectations and previously published studies [22, 24, 26, 28, 29, 35]. Of note, this study represents an early use of palbociclib in the NHS clinical setting; therefore, management of adverse events may have changed with increasing familiarisation with the treatment. This data adds to the growing body of real-world evidence demonstrating palbociclib effectiveness and tolerability (see Supplementary Table 5).

This study remains subject to limitations. First, patients were recruited from larger centres enrolled in the IPP, which may differ from smaller centres in terms of demographic and clinical characteristics, and therefore may not be representative of all patients treated with palbociclib in routine clinical practice as part of the IPP. Furthermore, differences in requirements for patient consent for living patients at different centres may have introduced bias in the interpretation of study outcomes. Second, common to all retrospective study designs, the interpretation of study outcomes is dependent on the completeness and quality of the medical records and the reliability of the abstraction of data from the medical records, although source data verification was employed to identify and correct any abstraction errors. Standard imputation of dates and menopause status were required in a few instances, which may have introduced bias in the interpretation of the study outcomes. Furthermore, confounding factors cannot be ruled out; although information regarding prior treatment history, disease severity and comorbidities (17% of the patient population had comorbidities [data not presented]) were collected, concomitant therapy was not. Thirdly, due to the nature of routine clinical practice in the UK, data on quality of life is not routinely collected, which means we are unable to compare outcomes reported by PALOMA-2. Furthermore, this study only reports on common adverse events from palbociclib treatment and therefore does not represent the true incidence of rarer adverse events in a real-world setting. Despite these limitations, this study provides important insights into the characteristics and clinical outcomes of the first cohort of patients with HR-positive/HER2-negative MBC treated with palbociclib according to routine clinical practice in centres across the UK prior to it being made widely available by NICE and SMC.

## Conclusions

This study demonstrates the effectiveness and tolerability of palbociclib within the first cohort of patients with HR-positive/HER2-negative advanced/MBC treated in routine clinical practice in the UK NHS prior to the funding agreement. It highlights that patients with de novo MBC appear to derive greater benefit than those with relapsed MBC and that delaying the initiation of palbociclib may compromise clinical efficacy. Patient access schemes like the IPP can bridge the gap between regulatory approval and NHS funding for new medicines, providing benefits to patients as well as facilitating the collection of data to evaluate real-world outcomes.

## Supplementary information

Supplemental material

## Data Availability

The datasets generated during and/or analysed during the current study are stored in Pfizer centralised repository and are available via a medical information data request.
