# Supplementary material for: A real-world study of the first use of palbociclib for the treatment of advanced breast cancer within the UK National Health Service as part of the novel Ibrance® Patient Program
Source: Br J Cancer. 2023 Jul 19;129(5):852–60. doi: 10.1038/s41416-023-02352-5 (PMC10449843; doi:10.1038/s41416-023-02352-5)
Supplement: Supplementary file 1 — Supplemental material [file 41416_2023_2352_MOESM1_ESM.pdf]

A real-world study of the first use of palbociclib for the treatment of advanced breast cancer within the UK National Health Service as part of the novel Ibrance® Patient Program

Authors

Carlo Palmieri[1,2], Alison Musson[3], Catherine Harper-Wynne[4], Duncan Wheatley[5], Gianfilippo Bertelli[6], Iain R Macpherson[7], Mark Nathan[8], Ellie McDowall[8], Ajay Bhojwani,[1,2], Mark Verrill[9], Joe Eva [10], Colm Doody[11], Ruhe Chowdhury[8,11,12]

Affiliations

1. The Clatterbridge Cancer Centre NHS Foundation Trust, Liverpool, UK
2. Department of Molecular and Clinical Cancer Medicine, University of Liverpool, Liverpool, UK.
3. The Christie NHS Foundation Trust, Manchester, UK.
4. Kent Oncology Centre, Maidstone and Tunbridge Wells NHS Trust, Kent, UK.
5. Department of Oncology, Royal Cornwall Hospitals NHS Trust, Truro, UK.
6. University Hospitals Sussex NHS Trust, Brighton, UK.
7. School of Cancer Sciences, University of Glasgow, Glasgow, UK.
8. Guy's and St Thomas' NHS Foundation Trust, London, UK.
9. Department of Medical Oncology, Northern Centre for Cancer Care, Freeman Hospital, Newcastle upon Tyne, UK.
10. OPEN Health, The Weighbridge, Brewery Courtyard, High Street, Marlow, UK.
11. Pfizer UK, Walton Oaks, Dorking Rd, Tadworth, UK.
12. Guys and St Thomas' NHS Trust, Great Maze Pond, London, SE1 9RT

Corresponding author

Professor Carlo Palmieri

The Clatterbridge Cancer Centre NHS Foundation Trust

Liverpool

UK

c.palmieri@liverpool.ac.uk

Journal name

British Journal of Cancer

Supplemental introduction

Table 1. Differences between the Ibrance Patient Program and the Palbociclib UK compassionate use program.

	Ibrance® Patient Program	Palbociclib UK compassionate access program [1]
**Licenced**	On label	Off label
**Line of therapy**	First-line	Fourth-line
**Number of patients**	191[a]	118
**Median Age (years)**	57	59
**Post-menopausal (%)**	70	82.2
**Outcomes**		
Progression free survival (months)	20.2	4.5
Overall survival (months)	Not reached	15.8
Overall response rate (%)	42	15.8

[a]First line patients n=137. The IPP revealed that clinicians used the scheme to access palbociclib for some patients outside of the first line setting and outside of the pre-specified criteria. These cases were a minority of the total cases.

Supplementary methods

Study design and data source

The Ibrance® Patient Program (IPP) was established to provide access to palbociclib pending National Institute for Health and Care Excellence (NICE) approval as per licenced indication in combination with an aromatase inhibitor (AI) for first-line treatment of United Kingdom (UK) National Health Service (NHS) patients with previously untreated, hormone receptor-positive//human epidermal growth factor 2-negative (HR-positive/HER2-negative) locally advanced or metastatic breast cancer (MBC). The IPP was open between April to December 2017. Patients were eligible for inclusion in the study if they enrolled into the IPP at one of the selected hospitals; gave written informed consent to access their medical records (where required); were aged ≥18 years at the time of enrolment into the IPP and received ≥1 dose of palbociclib as part of the IPP. Access to the IPP was via centres registering patients with Pfizer UK indicating they met the eligibility criteria. Palbociclib was provided to patients until they were discontinued by the treating clinical team. The IPP involved 116 NHS trusts, with 843 patients initiated palbociclib treatment via the IPP. This study reported on a subset (n=191) of patients enrolled into the IPP.

A UK, multi-centre, non-interventional cohort study to describe the real-world treatment patterns, patient characteristics, clinical outcomes and selected adverse events associated with palbociclib treatment for up to 2 years post-initiation in UK patients treated as part of the IPP. Study sites were selected based on the following criteria: 1. They were geographically dispersed across the UK, 2. Their cancer service participated in the IPP. 3. They had appropriate and sufficiently trained personnel available locally to identify eligible patients and support the study delivery in accordance with all applicable legal and regulatory requirements. 4. They treated at least 15 patients as part of the IPP. One site was selected due to the lead clinician's membership in the steering committee for the study. Membership of the steering committee was based upon two criteria: 1) membership in the National Cancer Research Institute (NCRI) MBC subcommittee and, 2) treatment of patients in the IPP. All members of the steering committee met these criteria. The NCRI subcommittee is an independent committee from Pfizer, and Pfizer had no input into the committee as part of this study. 5. They have an interest in taking part in the study. In total 9 NHS trusts were approached based on the above criteria, however only 8 NHS trusts fully met all the criteria for participation (the other site did not have enough resources for participation), these sites include: The Clatterbridge NHS Foundation Trust, Liverpool; The Christie NHS Foundation Trust, Manchester; Beatson Institute for Cancer Research, Glasgow; Freeman Hospital, The Newcastle upon Tyne Hospitals, NHS Foundation Trust, Newcastle; Kent Oncology Centre, Maidstone and Tunbridge Health NHS Trust; Guy's and St Thomas' NHS Foundation Trust, London; The Royal Cornwall Hospital, Cornwall; Brighton and Sussex University Hospitals NHS Trust. All patients enrolled into the IPP at these sites were included in this study unless they did not consent or were found to be ineligible. Data was collected retrospectively from patients' hospital medical records (paper-based or electronic, as appropriate) for up to 2 years post-initiation of palbociclib by trained representatives of the direct care team at each centre using electronic case report forms designed specifically for the study. Written informed consent from patients was not required at six centres, however, two centres had a local requirement for written informed consent from living patients. In the centres where consent was required, patients who did not consent to data collection from medical records were excluded from the study. The palbociclib initiation date was defined as the index date. The

baseline period was defined as the period from the date of diagnosis until the date of palbociclib initiation (index date). The post-initiation observation period was defined as the period from the index date until 2 years post-index or until the date of death or loss to follow-up, whichever was soonest. The study was approved by the NHS Health Research Authority and Health and Care Research Wales (London - Westminster Research Ethics Committee; reference: 18/LO/1859; October 2018).

Treatment line

First-line: First systemic MBC treatment, palbociclib started <3 months prior to initiation of AI. First-line added: Palbociclib added to ongoing AI, AI started >3 months prior to initiation of palbociclib. Second-line: Palbociclib plus AI given after at least one other treatment in the metastatic setting.

Study objectives and outcomes

The primary objective of the study was to describe patient baseline demographic and clinical characteristics at initiation of palbociclib; associated outcomes included: patient characteristics (age, sex, ethnicity, menopausal status, comorbidities); breast cancer disease characteristics (time since diagnosis, recurrence, stage, sites of metastases, oestrogen-receptor status, progesterone-receptor status, and HER2 status, and disease free interval [defined as the time since completion of prior (neo)adjuvant therapy (de novo metastatic; ≤12 months; >12 months) to recurrence]; breast cancer treatment history prior to palbociclib initiation.

Secondary objectives and associated outcomes included: palbociclib treatment patterns (starting dose, dose reductions, treatment interruptions and permanent treatment discontinuations, reasons for discontinuation, palbociclib treatment cycles and treatment duration at 1-, 2-years post-initiation); palbociclib clinical outcomes assessed as real-world timings (overall survival [OS] defined as the time from the date of palbociclib initiation until death from any cause as assessed by the individual centres [clinic visit, general practitioner records, etc]; progression-free survival [PFS] defined as the time from the date of palbociclib initiation to the date of first documented disease progression or death as assessed during a clinic visit; best overall response [complete response (CR), partial response (PR), stable disease, progressive disease] as assessed by the treating physician; time to best response and time to CR/PR at 1-, 2-years post-initiation); Selected adverse events (AE) overall and by grade during the first 12 months post-initiation were assessed from medical records, including: neutropenia (neutropenia grade was defined based on the absolute neutrophil count during the first 6 months post-initiation based on laboratory results [grade 1: 1.5 < 2.0 x 10^9/L, grade 2: 1.0 < 1.5 x 10^9/L, grade 3: 0.5 < 1.0 x 10^9/L, grade 4: < 0.5 x 10^9/L]), febrile neutropenia, diarrhoea, nausea and vomiting. AE were selected based on common AE observed in PALOMA-2 and 3, plus AE which are likely to affect patient quality of life. Furthermore, AE recorded for these patients was as a result of standard reporting to yellow card.

Additional analyses

Further subgroup analyses were carried out in pre-menopausal patients, to determine the effectiveness of palbociclib in this subset of patients. Time-to-event outcomes (PFS, OS and duration of treatment) were analysed and presented using the Kaplan-Meier method, with survival reported as median (95% confidence intervals [CI]) and/or 12- and 24-month survival rates).

Upon reviewing the PFS in the overall population we observed that some patients had a PFS of <2 months. Therefore, time to palbociclib discontinuation was assessed as a surrogate for PFS. Time to palbociclib discontinuation (Duration of treatment) was defined as the time from the date of palbociclib initiation to the date of palbociclib discontinuation and was analysed and presented using the Kaplan-Meier method, with discontinuation reported as median (95% confidence intervals [CI]) and/or 12- and 24-month discontinuation rates). Reasons for palbociclib discontinuation after documented disease progression was also captured (Presented in table 3 online resource 2), to determine the rationale behind treatment continuation in the clinical setting.

Supplementary Results

Table 2. Clinical outcomes of patients treated with palbociclib during 24 months of follow-up

	**Overall population**	**Palbociclib 1st line[a]**	**Palbociclib 1st line added to letrozole[b]**	**Palbociclib 2nd line[c]**	***De novo* MBC[d]**	**Non-*de novo* MBC[e]**	**Early relapse (<12 months)[f]**	**Late relapse (>12 months)[g]**
**Best response (24 months)[h]**, n (%)	**n=191**	**n=137**	**n=30**	**n=20**	**n=57**	**n=134**	**n=61**	**n=48**
Complete response	4 (2%)	3 (2%)	0 (0%)	1 (5%)	2 (4%)	2 (1%)	1 (2%)	1 (2%)
Partial response	76 (40%)	59 (43%)	10 (33%)	6 (30%)	26 (46%)	50 (37%)	20 (33%)	22 (46%)
Stable disease	91 (48%)	59 (43%)	16 (53%)	13 (65%)	25 (44%)	66 (49%)	30 (49%)	21 (44%)
Progressive disease	14 (7%)	12 (9%)	2 (7%)	0 (0%)	2 (4%)	12 (9%)	8 (13%)	2 (4%)
Not recorded	6 (3%)	4 (3%)	2 (7%)	0 (0%)	2 (4%)	4 (3%)	2 (3%)	2 (4%)
**Time to response (months)[h]**, median (range)	**n=185**	**n=133**	**n=28**	**n=20**	**n=55**	**n=130**	**n=59**	**n=46**
Time to best response	3.5 (0.1 - 23.8)	3.4 (0.2 - 23.8)	4.0 (0.6 - 18.6)	3.9 (0.1 - 10.4)	3.4 (0.1 - 19.7)	3.6 (0.2 - 23.8)	3.8 (0.2 - 23.8)	3.9 (1.0 - 23.2)
Time to first CR/PR	**n=80** 3.8 (0.1 - 23.7)	**n=62** 3.4 (0.2 - 23.7)	**n=10** 5.5 (1.7 - 18.6)	**n=7** 3.8 (0.1 - 10.4)	**n=28** 3.0 (0.1 - 19.6)	**n=52** 4.2 (1.7 - 23.7)	**n=21** 5.4 (2.5 - 23.7)	**n=23** 3.4 (2.1 - 23.1)
**Overall survival**	**n=190**	**n=136**	**n=30**	**n=20**	**n=56**	**n=134**	**n=61**	**n=48**
Median OS, months (95% CI)	NR	NR	NR	NR	NR	NR	NR	NR
12-month OS rate (95% CI)	86.3% (81.6% -	89.7% (84.7% -	76.7% (62.9% -	80.0% (64.3%	96.4% (91.7% -	82.1% (75.8% -	75.4% (65.3% -	83.3% (73.4% -

	91.3%)	95.0%)	93.4%)	- 99.6%)	100.0%)	88.8%)	87.0%)	94.6%)
24-month OS rate (95% CI)	71.5% (65.3% - 78.2%)	74.2% (67.1% - 81.9%)	70.0% (55.4% - 88.5%)	55.0% (37.0% - 81.8%)	82.1% (72.6% - 92.8%)	67.0% (59.5% - 75.5%)	55.6% (44.4% - 69.6%)	74.8% (63.4% - 88.2%)
**Progression-free survival[h]**	**n=191**	**n=137**	**n=30**	**n=20**	**n=57**	**n=134**	**n=61**	**n=48**
Median PFS, months (95% CI)	20.2 (14.7 - NR)[i]	22.8 (16.5 - NR)	11.8 (10.0 - NR)	7.8 (6.8 - NR)	NR	14.6 (11.4-22.3)	8.9 (4.4-12.2)	NR
12-month PFS rate (95% CI)	62.3% (55.8% - 69.6%)	68.6% (61.3% - 76.8%)	46.7% (31.8% - 68.4%)	40.0% (23.4% - 68.4%)	77.2% (67.0% - 88.9%)	56.0% (48.2% - 65.0%)	36.1% (25.8% - 50.4%)	75.0% (63.7% - 88.3%)
24-month PFS rate (95% CI)	45.5% (39.0% - 53.2%)	48.9% (41.2% - 58.0%)	36.7% (22.9% - 58.7%)	30.0% (15.4% - 58.6%)	59.6% (48.1% - 73.8%)	38.8% (31.4% - 48.0%)	21.3% (13.2% - 34.5%)	60.4% (48.1% - 76.0%)
**Time to palbociclib discontinuation**	**n=191**	**n=137**	**n=30**	**n=20**	**n=57**	**n=134**	**n=61**	**n=48**
Median, months (95% CI)	19.3 (13.9 - NR)	23.5 (14.1 - NR)	12.8 (10.4 - NR)	13.5 (7.4 - NR)	NR	14.4 (11.7 - NR)	10.3 (7.7 - 14.1)	NR
12-month rate (95% CI)	62.3% (55.8% - 69.6%)	65.0% (57.4% - 73.5%)	53.3% (38.2% - 74.5%)	55.0% (37.0% - 81.8%)	77.2% (67.0% - 88.9%)	56.0 (48.2% - 65.0%)	41.0% (30.3% - 55.4%)	72.9% (61.4% - 86.6%)
24-month rate (95% CI)	46.1% (39.5% - 53.7%)	49.6% (41.9% - 58.8%)	33.3% (20.1% - 55.3%)	35.0% (19.3% - 63.6%)	57.9% (46.4% - 72.2%)	41.0% (33.5% - 50.3%)	24.6% (15.8% - 38.2%)	58.3% (45.9% - 74.1%)

[a]Patients with no prior treatments prescribed in the metastatic setting prior to palbociclib initiation; [b]Patients with palbociclib added to ongoing letrozole, letrozole started > 3 months prior to the initiation of palbociclib; [c]Patients receiving palbociclib after at least one other treatment in the metastatic setting; [d]Patients diagnosed with *de novo* metastatic breast cancer; [e]Patients diagnosed with recurrent, metastatic breast cancer; [f]Patients with less than 12 months between last date of adjuvant endocrine therapy and diagnosis of metastatic breast cancer; [g]Patients with more than 12 months between last date of adjuvant endocrine therapy and diagnosis of metastatic breast cancer. Note: *De novo* MBC and non-*de novo* MBC groups are mutually exclusive; and early relapse (< 12 months), late relapse (>12 months) and *de novo* MBC groups are mutually exclusive. [h]7 instances of radiological assessment required standard imputation of dates (all 7 instances had only the month and year recorded). [i]In the overall population 18 (9%) patients had a PFS of less than 2 months. **Abbreviations:** CI, confidence interval; CR, complete response; MBC, metastatic breast cancer; NR, not reached; OS, overall survival; PFS, progression free survival; PR, partial response.

Figure 1: Kaplan–Meier plots of time to palbociclib discontinuation in the overall population and by treatment line (a) and patients with and without de novo MBC (b).

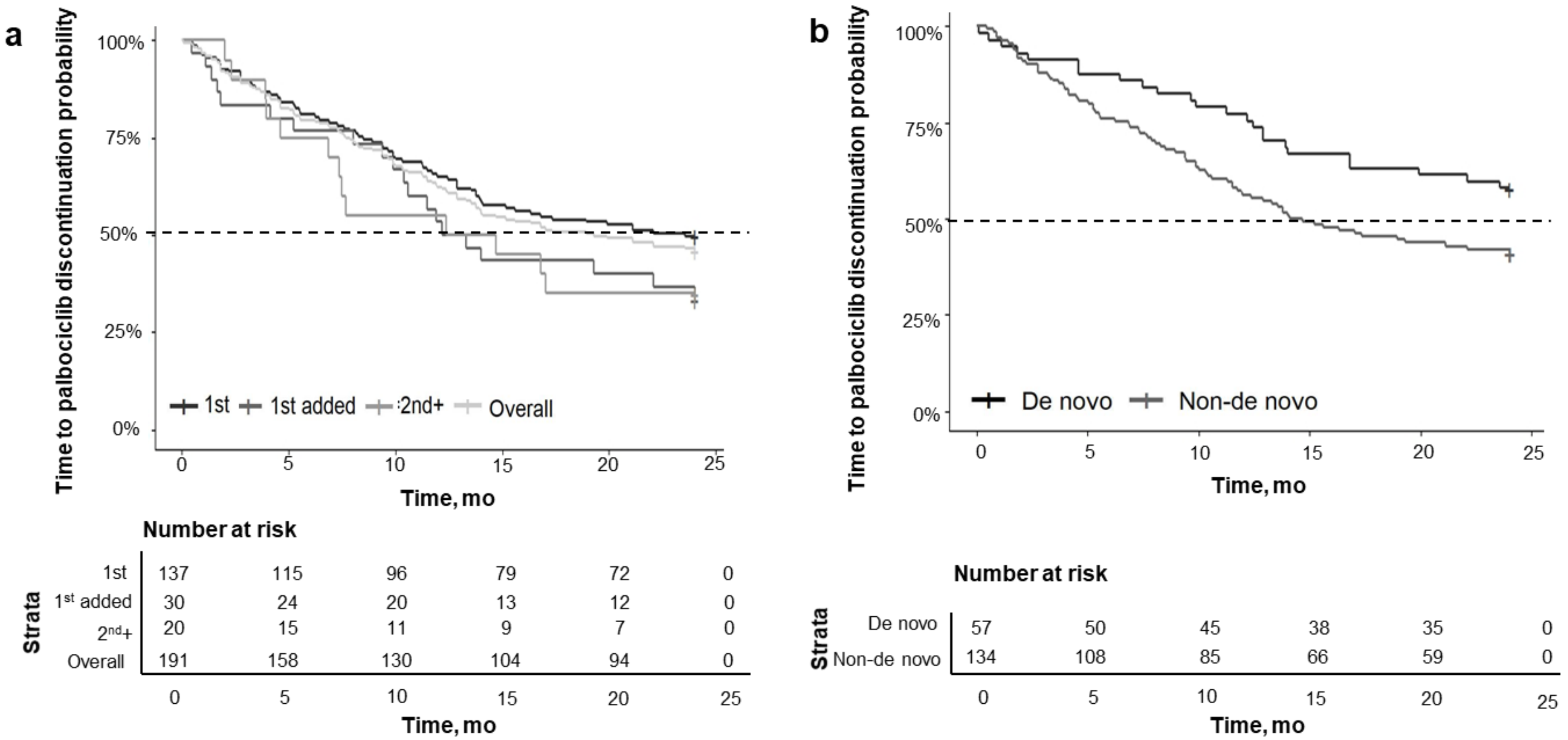

Definitions: 1st: First line, no prior treatments in the metastatic setting; 1st added: First-line added to letrozole, palbociclib added to ongoing letrozole, letrozole started >3 months prior to initiation of palbociclib; 2nd+: second or subsequent line of therapy; de novo: de novo MBC patients; Non -de novo: Non-de novo (relapsed) MBC patients; mo: months.

Table 3. Clinical outcomes of pre-menopausal patients treated with palbociclib during 24 months of follow-up

	**Pre-menopausal at MBC diagnosis (1st-line only)** [a, b] **n=34**
**Overall survival**	
Median, months (95% CI)	NR
12-month rate (95% CI)	97.1% (91.5%-100%)
24-month rate (95% CI)	70.6% (56.8%-87.7%)
**Progression free survival**	
Median, months (95% CI)	16.2 (12.4 – NR)
12-month rate (95% CI)	64.7% (50.5%-82.9%)
24-month rate (95% CI)	35.3% (22.4%-55.6%)
**Time to palbociclib discontinuation**	
Median, months (95% CI)	14.6 (9.9 – NR)
12-month rate (95% CI)	52.9% (38.6% - 72.7%)
24-month rate (95% CI)	35.3% (22.4% - 55.6%)

[a]Patients with no prior treatments prescribed in the metastatic setting prior to palbociclib initiation. [b]Pre-menopausal patients prescribed either LHRH or chemotherapy as per licence indication. **Abbreviations:** CI, confidence interval; MBC, metastatic breast cancer; NR, not reached; OS, overall survival; PFS, progression free survival.

Table 4. Reasons for continuation on palbociclib in individuals with documented progressive disease.

	Number of patients (%) [a]<br>n=39
Clinical decision not PD	11 (28%)
MDT decision not PD	6 (15%)
OPD and scan dates misaligned	3 (8%)
Initial PD response was a baseline scan	6 (15%)
PD clinic and OPA was after the scan	2 (5%)
Other [b]	3 (8%)
Not PD but no reason given	8 (21%)

[a] These patients were investigated due to them continuing treatment after documented progression. [b] Reasons include stop date incorrect - corrected now; Requested positron emission tomography (PET) scan to confirm given the small volume; was PD however time was taken to get the patient off treatment (most likely a clinic appointment and scan mismatch). Abbreviations: MDT, multidisciplinary team; OPA, outpatient appointment; PD, progressive disease.

Table 5: Summary of previously published real world evidence studies reporting patient demographics and real-world outcomes of patients with HR-positive/HER2-negative locally advanced or MBC treated with palbociclib.

Patient demographics										Effectiveness			Tolerability			Dose changes/discontinuations		
**Study**	**Year**	**Region**	**Number of patients**	**Median age (years [range])**	**Sex % female**	**% post-menopausal**	**Lines prior therapy in advanced metastatic setting**	**Endocrine backbone**	**Dosage**	**PFS (months; 95% CI)**	**OS (months; 95% CI)**	**ORR (%)**	**AE Grade 1-2 (%)**	**SAE grade 3-4 (%)**	**Type of AE or SAE**	**Reduction (%)**	**Interruption (%)**	**Discontinuation (%)**
DeMichele et al[2]	2021	USA	772	66 (58–73)	100	N/A	0	letrozole	N/A	1st line 20.0 (17.5–21.9)	NR, OS rate 78.3% at 24 months, 64.8% at 36 months	N/A	N/A	N/A	N/A	N/A	N/A	N/A
Rugo et al[3]	2022	USA	1324	67 (61-74)	99.2	100	0	AI	N/A	19.8 (17.9 - 21.7)	53.4 (48.7 – 58.6)		N/A	N/A	N/A	N/A	N/A	N/A
Current study	2022	Europe	191	57 (24.3–90.9)	99	70	0 to 1	Anastrozole Letrozole Exemestane, Fulvestrant	100-125 mg	Overall population 20.2 (14.7 - NR), 1st line 22.8 (16.5 - NR), 2nd line 7.8 (6.8 - NR)	NR OS rate 71.5% (overall population), 74.2% (1st line), 55.0% (2nd line) at 24 months	Overall population 42%, 1st line 45%, 2nd line 35%	N/A	N/A	Neutropenia (grade 3-4, 47%), febrile neutropenia (3%), diarrhoea (16%), nausea (18%), vomiting (11%)	41	40	54
Lin et al[4]	2021	USA	281	64.5	N/A	80.4	0 to 1	AI or fulvestrant	125 mg	1st line 21.2 (17.9–NR) 2nd line 11.5 (7.0-NR)	OS rate 71.4% (1st line) and 65.0% (2nd line) at 24 months	N/A	N/A	N/A	N/A	59.1 (reduced from 125-100mg)	N/A	33.5
Porte et al[5]	2020	Europe	310	61.8 (23.5-92.1)	100	81.6	0 to 1	Letrozole, Anastrozole, Exemestane, Fulvestrant LH-RH agonist	75-125 mg	Overall population 21.3 (17.5-25.2), 1st line 23.0 (20.8-NR), 2nd line 13.1 (9.0-18.6)	NR, OS rate 94.5% at 12 months, 81.8% at 24 months	N/A	N/A	N/A	neutropenia (grade 3-4, 72.3%), leukopenia (grade 3-4, 43.9%), anaemia (N/A grade 3-4, 3.2%), thrombocytopenia (grade 3-4, 2.9%)	29.4	N/A	5.7
Mycock et al[6]	2022	Japan	170	65.5 (31-90)	100	95.3	0 to >1	AI or fulvestrant	125 mg	PFS rate 73.3% (overall), 85.4% (1st line) and 56.4% (>2nd line) at 12 months. PFS rate 60.8% (overall), 66.5% (1st line) and 50.7% (>2nd line) at 24 months	OS rate 96.5% (overall), 99.0% (1st line) and 93.5% (>2nd line) at 12 months. OS rate 94.7% (overall), 99.0% (1st line) and 90.5% (>2nd line) at 24 months	N/A	N/A	N/A	N/A	31.2	N/A	33.5

Schneeweiss et al[7]	2020	Europe	1136	N/A	N/A	N/A	0 to 2	N/A	N/A	1st line 24.7 (11.9-NR), 2nd line 7.8 (5.8-15.4)	NR	N/A	N/A	<4	fatigue (11.3), nausea (11.3%), leukopenia or neutropenia (11.3%; grade 3-4 neutropenia 3.5%)	N/A	N/A	N/A
Waller et al[8]	2019	Other	162	64 (29-84)	100	100	0 to 2	Letrozole or fulvestrant	100-125 mg	PFS rate at 18 months was 80%	OS rate at 18 months was 88%	64.8	N/A	N/A	N/A	Palbo + letro (16), Palbo + Fulvest (5)	Palbo + letro (0), Palbo + Fulvest (2)	Palbo + Fulvest (14)
Petracci et al[9]	2020	Other	128	57 (29-84)	100	79.6	0 to >2	Letrozole, fulvestrant, exemestane, anastrozole	75 - 125 mg	Overall population 29.6 (19.5–38.8), 1st line 36.7 (18.1-42.6), 2nd and subsequent line 24.2 (12.0-32.7)	NR, OS rate 92.8% (1st line) and 74.0% (2nd line +) at 36 months	45.8	N/A	55.0	neutropenia (all grade 82%, grade 3-4; 20%) febrile neutropenia (7.6%), infection (27.5%)	15.2	46	2.0
Shao et al[10]	2021	Asia	81	54 (47–59)	100	77.8	0 to >2	AI or fulvestrant	125 mg	Overall population 10.8	N/A	N/A	N/A	N/A	N/A	N/A	N/A	N/A
Xi et al[11]	2019	USA	200	59.4 (50.7 – 67.7)	98	N/A	0 to >2	Letrozole or fulvestrant	N/A	1st line 20.7, 2nd line 12.8	N/A	N/A	N/A	N/A	Neutropenia (grade 1 - 4: 15.5%, 31.5%, 38.5%, 3% respectively), mucositis (4%), fatigue (3.5%), abscess (2%), nausea/vomiting (2%), diarrhoea (1.5%), rash (1.5%), elevated AST/ALT (1.5%), neuropathy (1.5%)	29	N/A	N/A
Mycock et al [12]	2022	Europe	1723	64 (30 – 90)	100	98	0 to ≥3	AI or fulvestrant	75 – 125 mg	Palbo + AI PFS rate 63.9% at 24 months. Palbo + Fulvest PFS rate 48.0% at 24 months	Palbo + AI OS rate 90.1% at 24 mnoths. Palbo + fulvest OS rate 88.6% at 24 months	Palbo + AI 84.5%, Palbo + fulvest 77.6%	N/A	N/A	N/A	Palbo + AI 18.4%, Palbo + fulvest 11.7%	N/A	Palbo + AI 19.7%, Palbo + fulvest 22.5%
Palumbo et al[13]	2021	Europe	182	62 (47–79)	100	73	0 to ≥3	Letrozole or fulvestrant	125 mg	Overall population 13 (3.2–25), 1st line 14 (9.5-25.0), 2nd line 11.7 (6.8-17.5), 3rd line + 6.7 (4.2–15.0)	Overall population 25 (12.5–32.4), 1st line (28.0), 2nd line (18.0), 3rd line + (13.0)	Overall population 34.6, 1st line 47.5%, 2nd line 31.3%, 3rd line+ 25.7%, de novo 52.2%	N/A	52.1	neutropenia (all grade 82.9%, grade 3–4 50.5%); febrile neutropenia (4.3%), mild nausea/vomiting (16.4%), alopecia (grade 1-2 12.7%), fatigue (grade 3 10.4%)	Palbo + Letro (14.5), Palbo + Fulvest (19.5)	Palbo + Letro (52.1), Palbo + Fulvest (44.5)	N/A
Bui et al[14]	2019	Europe	46	67 (35-85)	100	85	0 to ≥3	Letrozole or fulvestrant	125 mg	Overall population 10.0 (4.9-15.1)	N/A	N/A	78	13	Neutropenia (grade 3-4 63%)	22	9	4
Taylor-Stokes et al[15]	2019	USA	652	65.0 (30-90)	100	87.4	0 to ≥3	AI or fulvestrant	75 - 125 mg	Palbo + AI PFS rate 64.3% at 24 months	Palbo + AI OS rate 90.1% at 24 months	79.5	N/A	N/A	N/A	Palbo + AI (57.7), Palbo + Fulvest (14.4)	N/A	Palbo + AI (21.1), Palbo + Fulvest (19.9)

Kish et al[16]	2018	USA	763	64	100	N/A	0 to ≥3	letrozole	75 - 125 mg	N/A	N/A	N/A	N/A	N/A	Neutropenia (74.6%)	20.1	N/A	N/A
Demir et al[17]	2020	Asia	43	51 (44-58)	N/A	51.1	≥3	AI or fulvestrant	N/A	Overall population 7 (4-10)	11 (6-19)	13.9	N/A	N/A	Nausea (69.7%) Elevated transaminase levels (58.1%) Grade 3 neutropenia (27.9%), febrile neutropenia (4%)	14	N/A	N/A
Lee et al[18]	2020	Asia	169	57 (37-92)	N/A	97	0 to ≥4	Letrozole or fulvestrant	125 mg	Palbo + Letro 25.6 (19.1-NR) Palbo + Fulvest 6.37 (5.33-NR), De novo. NR	N/A	Palbo + Letro 39.6, Palbo + Fulvest 28.6	N/A	N/A	Neutropenia (93.1 or 95.8%), thrombocytopenia (31.7 or 50%)	Palbo + letro (51.0), Palbo + Fulvest (70.8)	N/A	N/A

Abbreviations: AE, adverse event; AI, aromatase inhibitors; ALT, alanine transaminase; AST, aspartate aminotransferase; CI, confidence interval; fulvest, fulvestrant; Letro, letrozole; LH-RH, luteinizing hormone – releasing hormone; N/A, not available; NR, not reached; NS, not specified; ORR, objective response rate; OS, overall survival; Palbo, palbociclib; PFS, progression-free survival; SAE, serious adverse event; USA, United States of America

References

1. Battisti NML, Kingston B, King J, Denton A, Waters S, Sita-Lumsden A, et al. Palbociclib and endocrine therapy in heavily pretreated hormone receptor-positive HER2-negative advanced breast cancer: the UK Compassionate Access Programme experience. *Breast Cancer Res Treat* **174** (3), 731–40 (2019).

2. DeMichele A, Cristofanilli M, Brufsky A, Liu X, Mardekian J, McRoy L, et al. Comparative effectiveness of first-line palbociclib plus letrozole versus letrozole alone for HR+/HER2- metastatic breast cancer in US real-world clinical practice. *Breast Cancer Res* **23** (1), 37–46 (2021).

3. Rugo HS, Brufsky A, Liu X, Li B, McRoy L, Chen C, et al. Real-world study of overall survival with palbociclib plus aromatase inhibitor in HR+/HER2- metastatic breast cancer. *NPJ Breast Cancer* **8** (1), 114-126 (2022).

4. Lin J, McRoy L, Fisher MD, Hu N, Davis C, Mitra D, et al. Treatment patterns and clinical outcomes of palbociclib-based therapy received in US community oncology practices. *Future Oncol* **17** (9), 1001–1011 (2021).

5. Porte B, Carton M, Lerebours F, Brain E, Loirat D, Haroun L, et al. Real life efficacy of palbociclib and endocrine therapy in HR positive, HER2 negative advanced breast cancer. *Breast* **54,** 303–310 (2020).

6. Mycock K, Zhan L, Hart K, Taylor-Stokes G, Milligan G, Atkinson C, et al. Real-world treatment patterns and clinical outcomes in patients receiving palbociclib combinations for HR+/HER2-advanced/metastatic breast cancer in Japan: Results from the IRIS study. *Cancer Treat Res Commun* **32,** 100573–100580 (2022).

7. Schneeweiss A, Ettl J, Lüftner D, Beckmann MW, Belleville E, Fasching PA, et al. Initial experience with CDK4/6 inhibitor-based therapies compared to antihormone monotherapies in routine clinical use in patients with hormone receptor positive, HER2 negative breast cancer - Data from the PRAEGNANT research network for the first 2 years of drug availability in Germany. *Breast.* **54,** 88–95 (2020).

8. Waller J, Mitra D, Mycock K, Taylor-Stokes G, Milligan G, Zhan L, et al. Real-World Treatment Patterns and Clinical Outcomes in Patients Receiving Palbociclib for Hormone Receptor-Positive, Human Epidermal Growth Factor Receptor 2-Negative Advanced or Metastatic Breast Cancer in Argentina: The IRIS Study. *J Glob Oncol*. **5,** JGO1800239-1800248 (2019).

9. Petracci F, Abuin GG, Pini A, Chacón M. RENATA study-Latin American prospective experience: clinical outcome of patients treated with palbociclib in hormone receptor-positive metastatic breast cancer-real-world use. *Ecancermedicalscience* **14,** 1058–1066 (2020).

10. Shao X, Zheng Y, Cao W, Shen X, Li G, Chen J, et al. Ki67 and progesterone receptor status predicts sensitivity to palbociclib: a real-world study. *Ann Transl Med* **9** (8), 707–718 (2021).

11. Xi J, Oza A, Thomas S, Ademuyiwa F, Weilbaecher K, Suresh R, et al. Retrospective Analysis of Treatment Patterns and Effectiveness of Palbociclib and Subsequent Regimens in Metastatic Breast Cancer. *J Natl Compr Canc Netw* **17** (2), 141–147 (2019).

12. Mycock K, Zhan L, Hart K, Taylor-Stokes G, Milligan G, Atkinson C, et al. Real-world treatment of patients with palbociclib for HR+/HER2-advanced/metastatic breast cancer: the Europe IRIS study. *Future Oncol*. **18** (3), 349–362 (2022).

13. Palumbo R, Torrisi R, Sottotetti F, Presti D, Rita Gambaro A, Collovà E, et al. Patterns of treatment and outcome of palbociclib plus endocrine therapy in hormone receptor-positive/HER2 receptor-negative metastatic breast cancer: a real-world multicentre Italian study. *Ther Adv Med Oncol* **13,** 1–18 (2021).

14. Bui TBV, Burgers DM, Agterof MJ, van de Garde EM. Real-World Effectiveness of Palbociclib Versus Clinical Trial Results in Patients With Advanced/Metastatic Breast Cancer That Progressed on Previous Endocrine Therapy. *Breast Cancer (Auckl)* **13,** 1–6 (2019).

15. Taylor-Stokes G, Mitra D, Waller J, Gibson K, Milligan G, Iyer S. Treatment patterns and clinical outcomes among patients receiving palbociclib in combination with an aromatase inhibitor or fulvestrant for HR+/HER2-negative advanced/metastatic breast cancer in real-world settings in the US: Results from the IRIS study. *Breast* **43,** 22–27 (2019).

16. Kish JK, Ward MA, Garofalo D, Ahmed HV, McRoy L, Laney J, et al. Real-world evidence analysis of palbociclib prescribing patterns for patients with advanced/metastatic breast cancer treated in community oncology practice in the USA one year post approval. *Breast Cancer Res* **20** (1), 37–44 (2018).

17. Demir A, Mandel NM, Paydas S, Demir G, Er Ö, Turhal NS, et al. Efficacy of Palbociclib and Endocrine Treatment in Heavily Pretreated Hormone Receptor-positive/HER2-negative Advanced Breast Cancer: Retrospective Multicenter Trial. *Balkan Med J* **37** (2), 104–107 (2020).

18. Lee J, Park HS, Won HS, Yang JH, Lee HY, Woo IS, et al. Real-World Clinical Data of Palbociclib in Asian Metastatic Breast Cancer Patients: Experiences from Eight Institutions. *Cancer Res Treat* **53** (2), 409–423 (2021).
